# Concentrated extract of Prunus mume fruit exerts dual effects in 3T3-L1 adipocytes by inhibiting adipogenesis and inducing beiging/browning

**DOI:** 10.29219/fnr.v65.5492

**Published:** 2021-10-29

**Authors:** Su Bu, Chunying Yuan, Fuliang Cao, Qifeng Xu, Yichun Zhang, Ronghua Ju, Longyun Chen, Zhong Li

**Affiliations:** 1 College of Biology and the Environment, Nanjing Forestry University, Nanjing, China; 2 Co-innovation Center for Sustainable Forestry in Southern China, Nanjing Forestry University, Nanjing, China; 3 College of Forestry, Nanjing Forestry University, Nanjing, China; 4 National Engineering Research Center of Biomaterials, Nanjing Forestry University, Nanjing, China; 5 Nanjing Longlijia Agricultural Development Co. Ltd., Nanjing, China; † These authors contributed equally to this study

**Keywords:** concentrated water extract of *Prunus mume* fruit (CEPM), beiging, browning, mitochondrial biogenesis, 3T3-L1 adipocytes

## Abstract

**Background:**

The fruit *Prunus mume* has beneficial effects in the treatment of obesity and metabolic syndrome. However, its mechanism of action is unclear.

**Objective:**

We assessed the effect of a concentrated water extract of *P. mume* fruit (CEPM) on adipogenesis and beiging/browning in 3T3-L1 cells.

**Methods:**

The cell viability was determined by MTT assay. Lipid accumulation was assessed with Oil Red O (ORO) staining under different concentrations of CEPM. The effects of CEPM treatment during differentiation on beiging/browning and mitochondrial biogenesis in 3T3-L1 cells were investigated.

**Results:**

CEPM treatment suppressed differentiation and decreased lipid accumulation by downregulating the expression of key adipogenic genes, including PPARγ, C/EBPα, SREBP-1c, FAS, and perilipin A. In contrast, CEPM treatment increased the mitochondrial DNA (mtDNA) content and mRNA levels of mitochondrial biogenesis genes, including *NAMPT*, *Nrf1*, *Nrf2,* and *CPT1*α, and reduced reactive oxygen species levels. Importantly, CEPM increased the expression of brown/beige hallmark genes (*Pgc-1*α, *Ucp1*, *Cidea*, *Cox7α1*, *Cox8b*, *Cd137*, and *Pdk-4*), as well as proteins (UCP1, PGC-1α, NRF1, TBX1, and CPT1α). The high-performance liquid chromatography (HPLC) analysis reveals that CEPM contains mumefural, naringin, 5-HMF, citric acid, caffeic acid, and hesperidin.

**Conclusion:**

The first evidence we provided showed that CEPM has a dual role in 3T3-L1 cells inhibiting adipogenesis and promoting beiging/browning, and hence, could be a potential agent in the fight against obesity.

## Popular scientific summary

- The concentrated water extract of *Prunus mume* fruit (CEPM) suppressed adipogenesis in 3T3-L1 adipocytes.
- CEPM stimulated mitochondrial biogenesis in 3T3-L1 adipocytes.
- CEPM increased the expression of key genes of beiging/browning in 3T3-L1 adipocytes.
- The bioactive components in CEPM, including flavonoids and phenolic acids, might contribute to the dual effects in 3T3-L1 adipocytes, and could be used as a potential agent in the fight against obesity.

The prevalence of obesity has risen dramatically in most countries in the world, which leads to a high risk of various diseases, including type 2 diabetes, cardiovascular disease, and certain types of cancers (1). It is mainly due to abnormal or excessive fat accumulation in adipose tissue, and occurs as a result of both adipocyte hyperplasia and hypertrophy (2).

Adipogenesis is regulated via a complex process involving coordinated changes in hormone sensitivity and gene expression regulated by specific transcription factors in preadipocytes, including CCAAT/enhancer binding proteins (CEBPs), peroxisome proliferator-activated receptor-gamma (PPARγ), and sterol-regulatory element binding proteins (SREBPs) (3). PPARγ and C/EBPα are the major transcription factors that regulate adipocyte differentiation (4). Activation of these factors can induce the expression of other adipogenesis-related transcription factors, such as SREBP-1c, to promote lipid accumulation by stimulating the expression of a cascade of downstream adipocyte-specific genes, including fatty acid synthase (FASN), acetyl CoA carboxylase 1 (ACC1), and perilipin A. This will eventually lead to differentiation of the preadipocytes into mature adipocytes (5). The *de novo* synthesis of fatty acids involves a number of key enzymes and proteins. Initially, ACC1 catalyzes the conversion of acetyl-CoA to malonyl-CoA to provide the substrate that is used for the fatty acid synthesis catalyzed by FASN. FASN is an important enzyme system that catalyzes the acyl transfer, dehydration, condensation, and reduction steps during the extension of fatty acid chains. After lipid droplets are formed, the lipid droplet coating protein perilipin A is expressed to prevent lipid droplets from being hydrolyzed by various enzymes in the cytoplasm.

The hydrolysis of triglycerides requires three lipases, namely, fatty triglyceride lipase (ATGL), hormone sensitive lipase (HSL), and monoacylglycerol lipase (MGL) (6) in sequence. The generated free fatty acids are then transported to the mitochondria for β-oxidation (7). Carnitine palmitoyltransferase 1-α (CPT1-α) is a key enzyme in fat metabolism, which effectively promotes the movement of long-chain fatty acids into mitochondria, thereby accelerating β-oxidation and the supply of metabolic energy to fat cells (8).

There are three types of adipose tissue: white adipose tissue (WAT), brown adipose tissues (BAT), and brown-like (beige) adipose tissue (9). Different from WAT, the function of which is to store energy, BAT and beige adipose tissues shared similar morphological and functional properties, such as multilocular lipid droplets and a high content of mitochondria expressing uncoupling protein 1 (UCP1) (10, 11). UCP1 is a transmembrane protein that is found in the inner membrane of mitochondria and allows for the dissipation of energy in the form of heat by uncoupling the mitochondrial proton gradient from adenosine triphosphate (ATP) synthesis, and is responsible for non-shivering thermogenesis (12, 13).

A key protein in regulating mitochondrial function is PGC1α. It acts as a positive regulator of mitochondrial biogenesis and respiration, adaptive thermogenesis, and gluconeogenesis, as well as many other metabolic processes (14, 15). As a co-activator of PPARγ, it therefore plays a central role in the regulatory network that controls mitochondrial biogenesis and respiratory function, interacting with multiple transcription factors to enhance oxidative phosphorylation in mitochondria, regulate the expression of UCP1 in BAT, and promote thermogenesis (16). The mitochondrial electron transport chain is the main site of reactive oxygen species (ROS) production in adipocytes (17). Browning of WAT can reduce the mitochondrial dysfunction induced by the accumulation of ROS.

Based on these findings, it is now believed that increasing the extent of adipose tissue browning or beiging could be a promising strategy for treating obesity and its related diseases. In this regard, numerous studies have shown that metabolites and bioactive components, such as flavonoids and polyphenol from plants, fruit, and vegetables, can very effectively induce the browning/beiging of white adipocytes.

*Prunus mume* (also called Ume in Japan and Maesil in Korea) has been widely cultivated as an ornamental natural plant (18). The fruit of *P. mume* contains various phenolic acids and flavonoids, which have been shown to exert an anti-diabetic effects both *in vitro* and *in vivo* (19), to have antioxidant and anti-osteoporosis actions (20), and to inhibit melanogenesis (21). Studies have reported that an extract of the *P. mume* leaf can reduce blood glucose levels in diabetic mice (22), stimulate glucose uptake, and ameliorate glucose intolerance and fat accumulation in mice fed a high-fat diet (19). In addition, a novel compound called mumefural, isolated from concentrated *P. mume* fruit juice can markedly improve blood fluidity and significantly improve blood circulation (23).

There have been limited studies, however, on adipocyte browning/beiging and the anti-obesity potential of *P. mume* extracts. In this study, we aimed to investigate the effects of a concentrated water extract of *Prunus mume* fruit (CEPM) on adipogenesis and beiging/browning in 3T3-L1 adipocytes, identify the major bioactive compounds, and elucidate the underlying molecular mechanism of action of CEPM.

## Materials and methods

### Materials

The CEPM (Longlijia, Nanjing) is a black, sticky paste, which is diluted to give a 1,000 mg/mL stock solution with Milli-Q water and stored at 4°C. 3T3-L1 preadipocytes were purchased from ATCC (CL-173, Manassas, VA, USA). Dulbecco’s modified Eagle medium (DMEM), fetal bovine serum (FBS), and 0.25% trypsin were purchased from Gibco (Rockville, MD, USA). 1 × Dulbecco’s Phosphate-Buffered Saline (DPBS), penicillin (100 U/mL), and streptomycin (100 µg/mL) solution (Pen-Strep) were purchased from Hyclone (Chicago, IL, USA). Insulin, dexamethasone (DEX), 3-isobutyl-1-methylxanthine (IBMX), Oil Red O (ORO), and 3-(4,5-dimethyl-2-thiazolyl)-2,5-diphenyl-2-H-tetrazolium bromide (MTT) were obtained from Sigma-Aldrich (St. Louis, MO, USA). The primary antibodies, anti-FASN, anti-perilipin A, anti-C/EBPα, anti-PPARγ, and anti-CPT1α, were acquired from Cell Signaling Technology (Danvers, MD, USA). Anti-SREBP 1c, anti-Pgc-1α, anti-UCP1, anti-Tbx1, anti-NRF1, and anti-cleaved PARP were acquired from Abcam (Cambridge, UK). Anti-β-actin, goat anti-mouse, goat anti-rabbit, and goat anti-rabbit- Fluorescein isothiocyanate (FITC) IgG secondary antibodies were obtained from Boster Biological Technology (Pleasanton, CA, USA). Mumefural was obtained from Zzbio Co. Ltd (Shanghai, China). 5-Hydroxymethylfurfural (5-HMF), caffeic acid, rutin, citric acid, quercetin, naringin, and hesperidin were obtained from Yuanye Bio-Technology Co. Ltd (Shanghai, China).

### High-performance liquid chromatography analysis

One gram of CEPM was accurately weighed into a 50-mL centrifuge tube and 40 mL of sterilized water was added. An ultrasonic pulverizer (300 W) was used for 40 min to completely dissolve the CEPM. The solution was then heated to 113°C and refluxed for 2 h. After cooling, the sample was filtered through a 0.45 μm filter membrane, and a 1 mL sample was placed into a high-performance liquid chromatography (HPLC) bottle for testing.

HPLC analysis was performed using a HPLC system (Alliance e2695, Waters, USA) equipped with a C18 (5 μm, 250 × 4.6 mm) column (X-Bridge, Waters, USA) at 35°C. The mobile phase consisted of solvent A (0.1% acetic acid in water) and solvent B (0.1% acetic acid in acetonitrile). The gradient conditions were as follows: initial 0 min, A:B (82:12, v:v); 0–15 min, A:B (78:22); 15–25 min, A:B (72:28), with a flow rate of 1.0 mL/min. Peaks were detected by measuring absorbance at 280 nm. A sample of 20 µL was injected into the column for analysis.

### Cell culture, differentiation, and CEPM treatment

The 3T3-L1 preadipocytes were differentiated as previously described (24). 3T3-L1 preadipocytes were propagated in DMEM (1 g/mL glucose) supplemented with 10% FBS and 1% Pen-Strep, and were maintained in a humidified atmosphere of 5% CO_2_ at 37°C. Two days post confluence, the 3T3-L1 preadipocytes were changed into differentiation-medium I (4.5 g/mL glucose DMEM, 10% FBS, 1% Pen-Strep, 1 µM DEX, 0.5 mM IBMX, and 10 µg/mL insulin) for 3 days to induce differentiation. Following this, the medium was replaced with differentiation-medium II (4.5 g/mL glucose DMEM, 10% FBS, 1% Pen-Strep, and 10 µg/mL insulin) for 2 days, and then maintained in differentiation-medium III (4.5 g/mL glucose DMEM, 10% FBS, 1% Pen-Strep) with a fresh medium change every 2–3 days until the end of differentiation. Different concentrations of CEPM were added along with differentiation-medium II until the end of differentiation unless otherwise specified.

Beige adipocyte differentiation was performed as previously described (25). Briefly, 2 days after confluence, the cells were treated with beige differentiation medium (DMEM containing 10% FBS, 0.5 mM IBMX, 0.5 μM dexamethasone, 1 μg/mL insulin, 50 nM T3, and 0.5 μM troglitazone) for 2 days (Days 2). Then, the medium was replaced with maintenance medium containing 1 μg/mL insulin, 50 nM T3, and 0.5 μM troglitazone once every 2 days (three times, days 2–8).

### MTT cell viability assay

3T3-L1 preadipocytes (100 μL containing 3 × 10^4^ cells) were added to each well of a 96-well plate in growth medium (1 g/mL glucose DMEM, 10% FBS, 1% Pen-Strep), and after culturing overnight, they were then treated with CEPM at different concentrations for 24 h. To investigate the effects of CEPM on mature adipocytes, the preadipocytes were treated with complete differentiation media for 10 days to ensure adipocyte differentiation and then treated with CEPM for 24 h in the maintenance media (DMEM supplied with 1% FBS and 1% Pen-Strep). Thereafter, 50 μL of the MTT solution (1 mg/mL) was added to each well and incubated for 4 h at 37°C. The cells were washed and replaced with 100 μL of dimethyl sulfoxide (Thermo Scientific, USA). The absorbance of the dissolved formazan was measured at 570 nm using a spectrophotometer (Synergy 2, BioTek, USA).

### ORO staining and lipid quantification

The cellular lipid content was assessed by ORO staining. The cells were fed with differentiation medium in the presence or absence of CEPM from Days 0 to 10, the mature adipocytes were washed twice with PBS, fixed in 10% formaldehyde for 1 h, and stained with an ORO working solution (0.6 mg/mL) for 1.5 h at room temperature. The stained cells were rinsed three times with sterilized, deionized water until no dye was released from the stained cells. The cells were photographed using an inverted fluorescence microscope (Ti2U, Nikon, Japan). The dye retained in the adipocyte was extracted with 200 μL of 100% isopropanol and quantified by measuring the absorbance of the extract at 510 nm. The results were expressed as relative lipid content compared with that of the control group without CEPM treatment.

### Mitochondrial biogenesis

For mitochondrial staining, 3T3-L1 preadipocytes were seeded into six-well plates and subjected to differentiation. Mito Tracker Green (Beyotime, Shanghai, China) was directly added to differentiation-medium III at a concentration of 100 nM, and the cells were kept in the dark for 30 min at 37°C in an atmosphere of 5% CO_2_. The stained mitochondria were photographed after imaging under a fluorescence microscope (Ti2U, Nikon, Japan).

The levels of intracellular ROS generated in differentiated adipocytes following 24 h of CEPM treatment were measured using a Reactive Oxygen Species assay kit (Beyotime Shanghai, China) following the manufacturer’s instructions. The fluorescent probe DCFH-DA (10 mM) was diluted 1,000-fold with serum-free DMEM and then added to the cells, which were incubated at 37°C for 20 min in the dark. After washing three times, the cells were visualized by fluorescence microscopy. The cells were then collected, and the fluorescent intensities were measured at an excitation wavelength of 488 nm and an emission wavelength of 525 nm by a microplate reader. The result was normalized to the protein content.

### Immunofluorescence

For immunostaining, the adipocytes were washed three times with PBS, then fixed with 4% paraformaldehyde for 20 min, and washed three times with PBS. Following this, the cells were permeabilized with 0.1% Triton X-100 (diluted in 1× PBS) for 10 min, and washed three more times with PBS. The permeabilized cells were blocked with 5% *Bovine serum albumin* (BSA) (Solarbio, Beijing, China) in PBS for 1 h. Then the samples were incubated with the anti-UCP1 antibody diluted 1:200 in PBS containing 1% BSA at 4°C overnight. After washing three times with PBS, the cells were incubated with the FITC-conjugated anti-goat antibody (1:200) in 1% BSA for 1 h at room temperature in the dark and then examined by fluorescence microscopy. Photographs of the fluorescent cells were then obtained.

### RNA isolation and qRT-PCR analysis

Total RNA was extracted using a MiNiBEST Universal RNA Extraction kit (TaKaRa, Otsu, Shiga, Japan), and the concentration of the total RNA obtained was measured using a Nanodrop spectrophotometer (Thermo Scientific, Waltham, MA, USA). A total of 500 ng of mRNA was reverse-transcribed into cDNA using a PrimeScript™RT Master Mix (Perfect Real Time) kit (TaKaRa, Otsu, Shiga, Japan), The quantitative real time PCR (qRT-PCR) of genes involved in lipid metabolism and browning was performed using an iTaq^TM^ Universal SYBR^®^ Green Supermix (Bio-RAD, CA, USA) in StepOnePlus Real-Time PCR System (Applied Biosystems Inc, Foster City, CA, USA). The qRT-PCR was also used to determine the relative mitochondrial copy number by calculating the expression ratio of the mitochondria-encoded gene Cytochrome b (Cytb, mtDNA) to the nuclear-encoded gene ribosomal protein large p0 (Rplp0, ntDNA). Target gene mRNA levels were normalized using the 2^–ΔΔCT^ method. Primers were purchased from Tsingke (Nanjing, China), and the sequences are provided in Table 1.

**Table 1:** Primers used for quantitative real time-PCR

Target gene	Accession no.	Primer sequence
*PPARγ*	NM_001308354.1	Forward: 5’- AGGCCGAGAAGGAGAAGCTGTTG-3’
		Reverse: 5’- TGGCCACCTCTTTGCTCTGCTC -3’
*C/EBPα*	NM_001308354.1	Forward: 5’- GCCCCCGTGAGAAAAATGAAG -3’
		Reverse: 5’- GAGGTGCGAAAAGCAAGGGA -3’
*FASN*	NM_007988.3	Forward:5’- ATTCGGTGTATCCTGCTGTC -3’
		Reverse: 5’- GCTTGTCCTGCTCTAACTGG -3’
*SREBP-1c*	NM_001278601	Forward: 5’- TGGACTACTAGTGTTGGCCTGCTT-3’
		Reverse: 5’- ATCCAGGTCAGCTTGTTTGCGATG-3’
*CPT1α*	NM_013495	Forward: 5’- GGGCTTGGTAGTCAAAGGCT -3’
		Reverse: 5’- TGCCTGTGTCAGTATGCCTG -3’
*Perilipin A*	NM_001113471.1	Forward: 5’- CACTCTCTGGCCATGTGGA -3’
		Reverse: 5’- AGAGGCTGCCAGGTTGTG -3’
*Pgc-1α*	XM_006503779.3	Forward: 5’-CCCTGCCATTGTTAAGACC-3’
		Reverse: 5’-TGCTGCTGTTCCTGTTTTC-3’
*UCP1*	XM_021170845.1	Forward: 5’-ACTGCCACACCTCCAGTCATT-3’
		Reverse: 5’-CTTTGCCTCACTCAGGATTGG-3’
*Cox7A1*	NM_009944.3	Forward: 5’-AGCTCTTCCAGGCCGACAAT-3’
		Reverse: 5’-GAGTCAGCGTCATGGTCAGT-3’
*Cox8B*	NM_007751.3	Forward: 5’-AGCCAAAACTCCCACTTCC -3’
		Reverse: 5’-TCTCAGGGATGTGCAACTTC-3’
*CD137*	NM_001077508.1	Forward: 5’-GGTCTGTGCTTAAGACCGGG-3’
		Reverse: 5’-TCTTAATAGCTGGTCCTCCCTC-3’
*Pdk-4*	NM_013743.2	Forward: 5’-CTGGACTTTGGTTCAGAAAATGCCT-3’
		Reverse: 5’-AGCGGTCAGGCAGGATGTCAAT-3’
*Cidea*	XM_021150964.1	Forward: 5’-TGCTCTTCTGTATCGCCCAGT-3’
		Reverse: 5’-GCCGTGTTAAGGAATCTGCTG-3’
*Dio-2*	NM_010050.3	Forward: 5’-CTTCCTCCTAGATGCCTACAAAC-3’
		Reverse: 5’-GGCATAATTGTTACCTGATTCAGG-3’
*Cytb*	XM_032895473.1	Forward: 5’-ACCTCCTATCAGCCATCCCA-3’
		Reverse: 5’- AGCGAAGAATCGGGTCAAGG-3’
*Rplp0*	XM_021162425.1	Forward: 5’-TGACATCGTCTTTAAACCCCG-3’
		Reverse: 5’- TGTCTGCTCCCACAATGAAG-3’
*Nrf1*	XM_030255219.1	Forward: 5’-CGAAAGAGACAGCAGACACG-3’
		Reverse: 5’-TTGAAGACAGGGTTGGGTT-3’
*Nrf2*	XM_021209307.2	Forward: 5’-AGAACAAGTGACAAGATGGGC-3’
		Reverse: 5’-TCGGTCATGCTGAATTCCTTC-3’
*Nampt*	NM_021524.2	Forward: 5’-GAATGTCTCCTTCGGTTCTGG-3’
		Reverse: 5’-TCAGCAACTGGGTCCTTAAAC-3’
*β-Actin*	NM_007393	Forward: 5’-GTTGGAGCAAACATCCCCCA-3’
		Reverse: 5’-ACGCGACCATCCTCCTCTTA-3’

### Immunoblot analysis

The 3T3-L1 cells were first washed with PBS, trypsinized, and pelleted by centrifuging at 800 rpm for 5 min at 4°C. The cell pellets were then lysed directly in RIPA Q lysis buffer supplemented with phosphatase and protease inhibitors (Biosharp, Anhui, China) for 5 mins on ice. After a brief sonication, the supernatant was collected by centrifuging at 12,000 rpm for 10 min at 4°C. The protein concentration was determined using a BCA Protein Quantitation kit. A total of 30 mg of the crude protein samples was separated by electrophoresis on 10% SDS-PAGE gels, and then transferred to polyvinylidene difluoride (PVDF) membranes (Bio-Rad, CA, USA). The internal reference protein, β-actin, was blocked with 5% skim milk powder in Tris-buffered saline containing 0.05% Tween 20 (TBST) for 1 h at room temperature to prevent non-specific binding. Blocking of all the other target proteins was performed with 5% BSA. Membranes were then incubated with specific primary antibodies at 4°C overnight according to the dilution-fold suggested by manufacturer. After three washes with TBST, the membranes were incubated with the appropriate secondary antibody for 1 h at room temperature, and the immunoreactive bands detected with Clarity^TM^ Western ECL Substrate (Bio-Rad, CA, USA) by chemiluminescence. The signals were quantified by densitometry with Image Lab software (Bio-Rad, CA, USA).

### Statistical analysis

The results were expressed as mean ± standard deviation (SD). Statistical analysis was performed using one-way ANOVA followed by post-hoc test, and *P*-values <0.05 (set at*) were considered to be statistically significant between groups.

## Results

### Analysis of the major compounds in CEPM

**Fig. 1:**
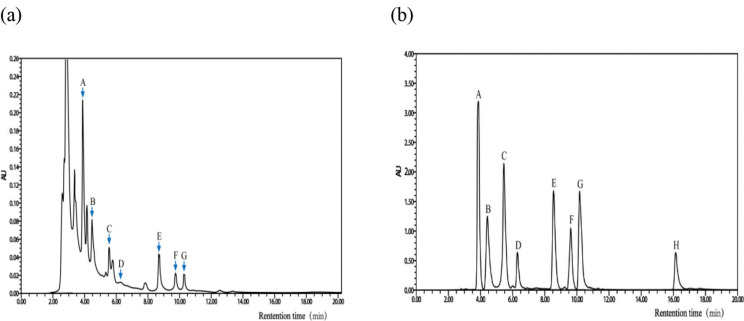
High-performance liquid chromatography (HPLC) analysis of the major compounds in CEPM. (a) HPLC analysis of standards, including (A) 5-hydroxymethylfurfural, (B) mumefural, (C) caffeic acid, (D) rutin, (E) citric acid, (F) naringin, (G) hesperidin, and (H) quercetin. (b) HPLC analysis of CEPM showing the presence of (A) 5-hydroxymethylfurfural, (B) mumefural, (C) caffeic acid, (D) rutin, (E) citric acid, (F) naringin, and (G) hesperidin.

In this study, we first identified several main components and their levels in CEPM by HPLC. First, the retention times of the standard flavonoids, 5-hydroxymethylfurfural, mumefural, caffeic acid, rutin, citric acid, naringin, hesperidin, and quercetin, were determined. Based on these retention times, we were able to identify six major components in CEPM that corresponded to the standards, and these were quantified as shown in Fig. 1 and Table 2. The levels were as follows: 5-HMF (0.58 ± 0.01 mg/g), mumeferal (0.92 ± 0.01 mg/g), naringin (0.63 ± 0.08 mg/g), citric acid (0.41 ± 0.02 mg/g), caffeic acid (0.19 ± 0.02 mg/g), and hesperidin (0.06 ± 0.02 mg/g).

**Table 2:** Levels of major flavonoids in CEPM

Peaks	Chemicals	CEPM (mg/g)
A	5-HMF	0.58 ± 0.01
B	Mumefural	0.92 ± 0.01
C	Caffeic acid	0.19 ± 0.02
E	Citric acid	0.41 ± 0.02
F	Naringin	0.63 ± 0.08
G	Hesperidin	0.06 ± 0.02

*Note*. All measurements were performed in triplicate. Data are expressed as mean ± SD.

### Effect of CEPM on cell viability in 3T3-L1 preadipocytes and adipocytes

**Fig. 2:**
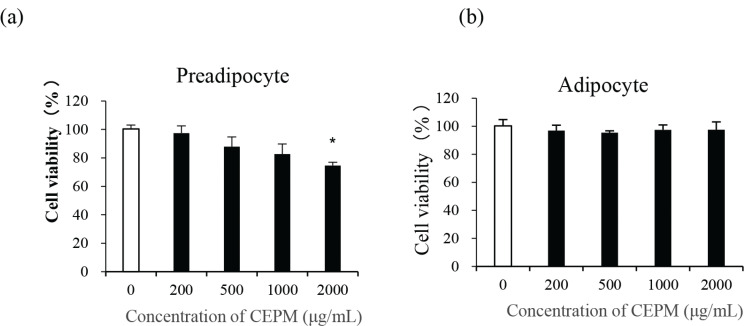
The effect of CEPM on the viability of 3T3-L1 cells. (a) The cell viability of preadipocytes treated with different concentrations of CEPM for 24 h. (b) The cell viability of adipocytes treated with different concentrations of CEPM for 24 h. Data are presented as the mean ± SD from three independent experiments. *Significant difference at *P* < 0.05.

An MTT assay was performed to assess whether CEPM treatment affects cell viability in 3T3-L1 cells. 3T3-L1 preadipocytes and differentiated adipocytes were treated with various concentrations of CEPM (200, 400, 500, 1,000, or 2,000 μg/mL) for 24 h, and cell viability relative to the control (as a percentage) was calculated. In preadipocytes, cell viability decreased from 2.7 to 25.4% in a dose-dependent manner, although, with the exception of 2,000 μg/mL CEPM, these decreases were not statistically significant (Fig. 2a). In contrast, there were only very modest decreases in the viability of differentiated adipocytes (ranging from 8.1 to 9.5% at concentrations of 200-2,000 μg/mL); however, these were not statistically significant compared with the control (Fig. 2b).

### CEPM inhibits adipogenesis in 3T3-L1 cells

**Fig. 3:**
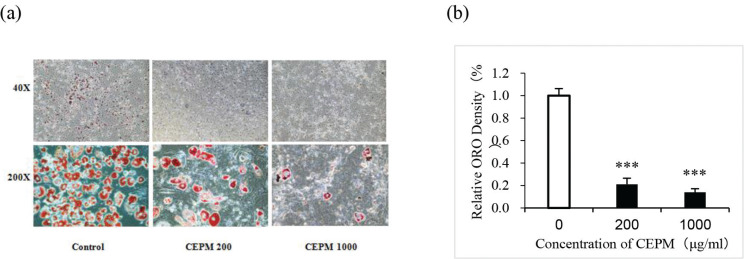
The effect of CEPM on lipid accumulation in 3T3-L1 cells. (a) Oil Red O staining of 3T3-L1 cells treated with or without CEPM during differentiation (Day 0–10) was performed on Day 10. (b) The ORO-stained lipid droplets were extracted using isopropanol, and the absorbance of the extract was determined at 510 nm. Data are presented as mean ± SD from three independent experiments. ***Significant difference at *P* < 0.001.

To evaluate the effect of CEPM on differentiation and lipid accumulation in 3T3-L1 cells, the lipid droplets in cells treated with or without CEPM during differentiation (Day 0–11) were stained with ORO dye after the differentiation process (Day 11) and photographed with reverse microscopy. From the microscopy analysis, it was found that the size and number of stained lipid droplets in the CEPM cells were significantly decreased compared with the control cells (Fig. 3a). Semi-quantification of lipid accumulation was achieved by dissolving the ORO dye in isopropanol and measuring the absorbance of the extract at 510 nm. This analysis also showed that cells treated with 200 and 1,000 μg/mL CEPM reduced the accumulation of intracellular lipid by 79 and 86%, respectively, compared with control cells (*P* < 0.001, Fig. 3b). This result suggests that CEPM inhibits differentiation and lipid accumulation in 3T3-L1 cells.

### Effect of CEPM on the expression of lipid metabolism related genes

**Fig. 4:**
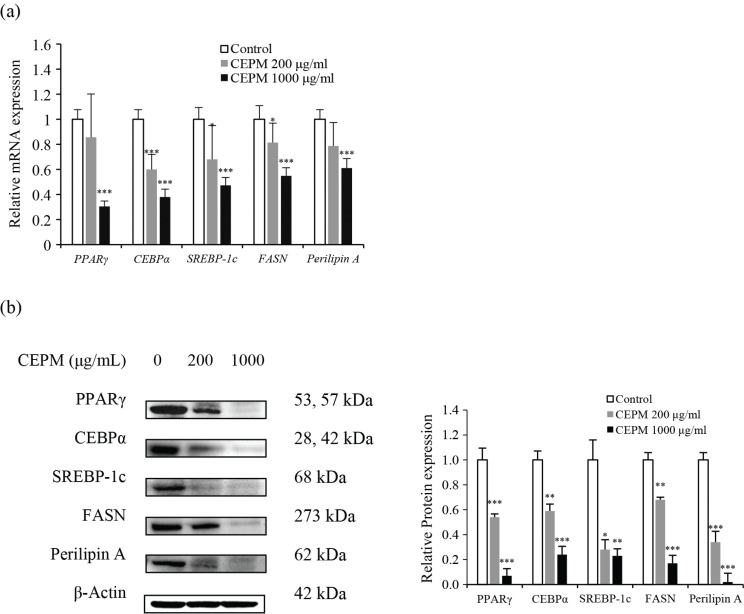
Effect of CEPM on the mRNA and protein expression of adipogenic genes. (a) The mRNA and (b) the protein expression levels of three adipogenic transcription factors and two key adipose proteins in 3T3-L1 adipocytes treated with different concentrations (200, 1,000 μg/mL) of CEPM during differentiation for 8 days. The mRNA levels were measured by a semi-quantitative polymerase chain reaction, and the protein levels were determined by semi-quantitative Western blotting. Data are presented as mean ± SD from three independent experiments. *Significant difference at *P* < 0.05, **Significant difference at *P* < 0.01, ***Significant difference at *P* < 0.001.

PPARγ is an essential transcription factor at the end of adipocyte differentiation. If this gene is knocked out, cells will not be able to differentiate into mature adipocytes. At the same time, multiple transcription factors of the C/EBP family play an important role in lipid metabolism. C/EBPβ is first activated in the early and middle stages of adipocyte differentiation, followed by the expression of C/EBPα and PPARγ. In addition, PPARγ activity is positively regulated by the transcription factor SREBP-1c. The expression of these three transcription factors eventually triggers the expression of a large number of downstream adipogenic genes, and the adipocyte differentiation process enters into its final stage. To directly examine the effect of CEPM on adipocyte differentiation, preadipocytes were treated with CEPM in differentiation-medium II, and the cells were then harvested at the end of differentiation. The mRNA levels of PPAR-γ, C/EBP-α, and SREBP-1c were all decreased by 14, 40, and 32%, respectively, upon treatment with 200 μg/mL CEPM, although only difference for C/EBP-α was significant (Fig. 4a, *P* < 0.001). In contrast, the mRNA expression levels of these three transcription factors decreased by 70, 62, and 53%, respectively, following treatment with 1,000 μg/mL CEPM, compared with control cells (Fig. 4a, *P* < 0.001).

In this study, we also examined the effect of CEPM on the mRNA expression levels of a number of key proteins that plays important roles in mature adipocytes. These included, FASN, a rate-limiting enzyme in the long-chain fatty acid extension process, and perilipin A, which surrounds the lipid droplets protecting them from hydrolysis by intracellular lipases, After CEPM treatment, the mRNA levels of *FASN* and *perilipin A* were decreased by 19 and 21%, as well as by 45 and 39% following treatment with 200 and 1,000 μg/mL CEPM treatment, respectively, compared with control cells (Fig. 4a, *P* < 0.001). The inhibitory effect of the high CEPM concentration was more obvious than that at the low concentration (Fig. 4a). Consistent with the qRT-PCR results, the protein levels of these three transcription factors and two adipogenic genes were all reduced in a dose-dependent fashion following treatment with CEPM, compared with control cells (Fig. 4b, *P* < 0.001). These findings indicate that CEPM treatment can dramatically inhibit adipogenesis-associated gene expression and suppress adipogenesis in 3T3-L1 adipocytes.

### CEPM stimulates mitochondrial biogenesis

**Fig. 5:**
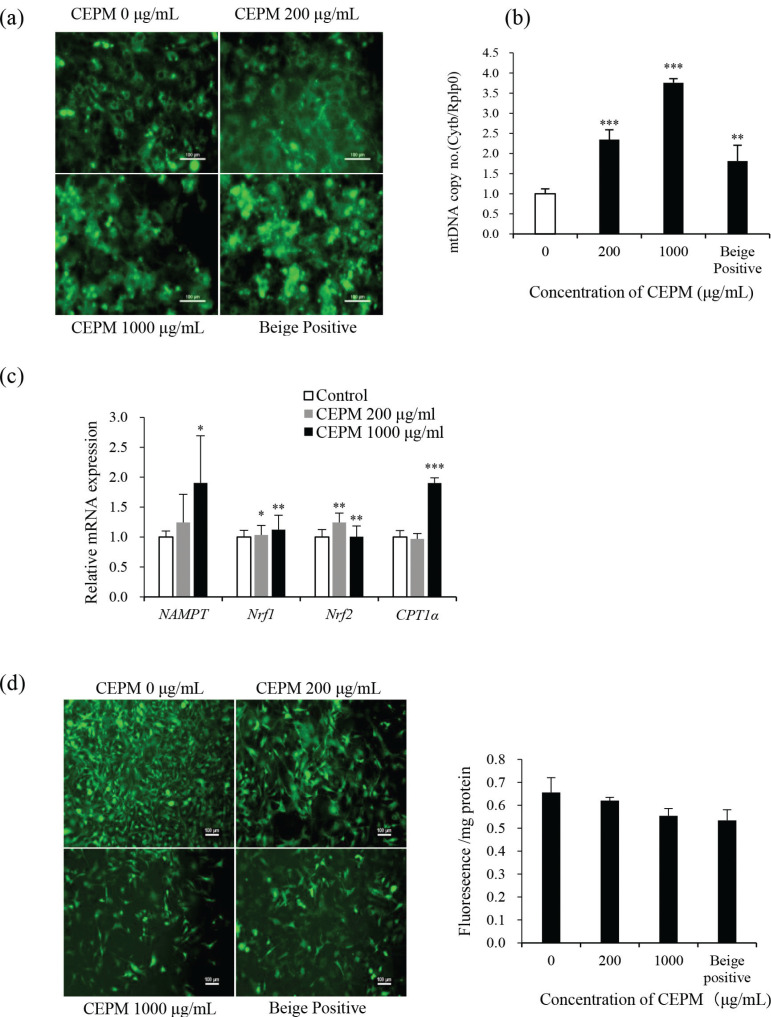
Effect of CEPM on mitochondrial biogenesis in 3T3-L1 adipocytes. (a) Distribution of adipocyte mitochondria assessed by MitoTracker green staining (200× magnification, scale bar = 100 μm). (b) The mtDNA copy number in preadipocytes (c) Relative mRNA expression levels of *Nampt, Nrf1, Nrf2,* and *CPT1*α assessed by real-time qPCR. (d) Intracellular ROS levels as detected by the DCFH-DA Reactive Oxygen Species assay kit (100× magnification, scale bar = 100 μm). Data are shown as mean ± SD of three independent experiments with three replicates each (*n* = 3). *P < 0.05, **P < 0.01, ***P < 0.001 versus control.

The transformation of white adipocytes into beige/brown adipocytes is often accompanied by an increase in mitochondrial biogenesis, which is involved in increased heat production and energy consumption. To evaluate this, we first performed an assessment of mitochondrial mass using Mito Tracker Green, a fluorescent stain that is selective of mitochondria. As shown in Fig. 5a, the mitochondrial network changed from dot-like structures that surrounded lipid droplets in mature adipocytes to being a multilocular pattern in adipocytes treated with CEPM. In addition, treatment with CEPM (both concentrations) resulted in higher fluorescence intensities than in control cells. In this experiment, we also included cells that were differentiated with beige adipocyte differentiation medium as a positive control. These cells also had a significantly higher green fluorescence intensity, suggesting that they contained more mitochondria. In addition, the effect of CEPM on mtDNA copy number was determined using qPCR to calculate the ratio of mtDNA to nuclear DNA (Cytb/Rplp0) in 3T3-L1 adipocytes. The qPCR results demonstrated that the copy number of mitochondrial DNA (mtDNA) in adipocytes increased during differentiation by 2.35- and 3.76-fold following treatment with 200 and 1,000 μg/mL of CEPM, respectively (Fig. 5b, *P* < 0.001).

**Fig. 6:**
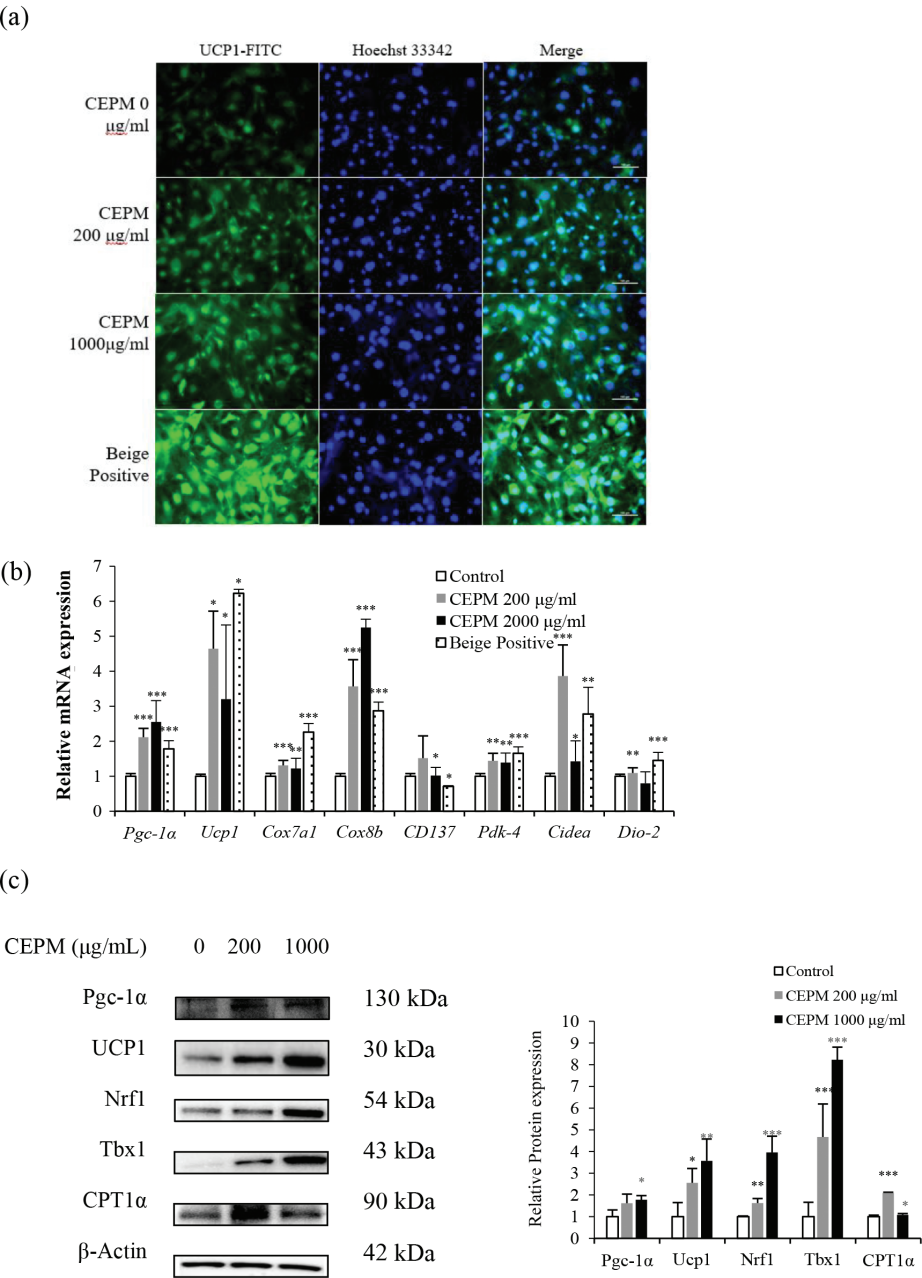
Effects of CEPM on browning/beiging in 3T3-L1 adipocytes. (a) The expression of UCP1 in 3T3-L1 adipocytes was evaluated by immunofluorescent staining (100× magnification, scale bar = 100 μm). (b) The expression of brown/beige marker genes in 3T3-L1 cells treated with CEPM at different concentrations for 10 days during differentiation or the beige positive control. (c) Western blotting of several browning marker proteins. Data are presented as mean ± SD from three independent experiments. *P < 0.05, **P < 0.01, ***P < 0.001.

In addition, genes related to mitochondrial biogenesis were upregulated upon CEPM treatment. For example, *NAMPT* levels were increased by 1.24- and 1.9-fold, respectively, following treatment with 200 and 1,000 μg/mL CEPM. *Nrf1* levels were not changed significantly at a low concentration, but were increased by 1.12-fold with 1,000 μg/mL CEPM treatment. Interestingly, the relative mRNA expression of *Nrf2* were increased 1.24-fold at a low concentration but were unchanged at a high concentration (Fig. 5c). CPT1α is a mitochondrial protein, which allows for the transport of synthesized long-chain fatty acids into mitochondria so that they can be degraded via β-oxidation. The mRNA expression of *CPT1α* was significantly increased by 90% following treatment with 1,000 μg/mL CEPM (Fig. 5c). Interestingly, the expression of CPT1α protein was increased to 209 and 108% upon treatment with 200 and 1,000 μg/mL of CEPM, respectively (Fig. 6c).

Mitochondria are generally considered to be the ‘powerhouse units’ of the cell. More than 95% of ATP required for cell metabolism and some other cellular processes are produced by mitochondria, and the mitochondrial electron transport chain is the main site of ROS generation in adipocytes. Excessive ROS accumulation can, however, induce mitochondrial dysfunction. Fluorescence microscopy showed that CEPM significantly scavenged mitochondrial ROS in a dose-dependent manner (Fig. 5d).

These data indicate that CEPM treatment of 3T3-L1 cells can upregulate the expression of mitochondrial biogenesis-related genes, increase the mitochondrial content, and improve the mitochondrial ability to clear ROS.

### CEPM induces adipocyte browning markers

It is well known that inducing the expression of UCP1, a protein associated with mitochondrial uncoupling, is a key feature in the transition from white adipocytes to brown adipocytes. We used immunofluorescent staining of cells treated with CEPM and observed a robust increase in UCP1-positive adipocytes (Fig. 6a). We also assessed the expression levels of UCP1 at the mRNA and protein levels by qPCR and Western blotting. The *Ucp1* mRNA expression levels were elevated by 4.6- and 3.2-fold by 200 and 1,000 μg/mL CEPM treatment, compared with control cells (Fig. 6b, *P* < 0.05). Moreover, UCP1 protein levels were increased in a dose-dependent manner, being 2.56- and 3.57-fold the control levels, respectively, after treatment with 200 and 1,000 μg/mL of CEPM (Fig. 6c, *P* < 0.05).

Next, we examined the expression of other markers specific for browning. Compared with the white adipocyte differentiation control group, CEPM significantly increased the mRNA expression levels of typical brown fat markers, including *Pgc-1α*, *Cox7α1*, *Cox8b*, and some beige adipocyte-specific markers of the TNF receptor superfamily, member 9 (*Tnfrsf9*, also known as *Cd137*), *Pdk-4*, as well as *Cidea,* except for *Dio2* (Fig. 6b). Among these, the protein expression levels of PGC-1α, NRF1, TBX1, and CPT1α also showed a significant upregulation (Fig. 6c).

## Discussion

Obesity is an abnormal condition caused by the excessive accumulation of lipids in WAT and arises as an increase in the number and size of mature adipocytes as well as a lack of BAT, the energy-consuming adipose tissue. Therefore, increasing BAT mass and/or activity and decreasing WAT mass are promising strategies to treat obesity and metabolic diseases.

Evidence has shown that many natural components, such as kaempferol, zeaxanthin, ginsenoside, laminaria japonica, and sulforaphene, can prevent and treat obesity by inhibiting adipogenesis and promoting lipolysis in adipocytes (26–29). *Prunus mume* fruits are traditionally used as preserves, pickled side dishes, etc. in East Asia. Later, the extract of *Prunus mume* fruits is widely used as a functional food in Japan, Korea, and China for its beneficial health effects, such as blood fluidity improvement, and antioxidant and anti-diabetes effects (19, 21, 23). In this study, we first report that CEPM can not only inhibit adipogenesis but also promote browning/ beiging and mitochondrial biogenesis in 3T3-L1 adipocytes.

In support of these findings, we found that the expression of transcription factors responsible for the mid-terminal stages of differentiation and their downstream target adipogenic genes, such as FASN and perilipin A, were all significantly inhibited by CEPM. However, the mRNA or protein levels of two lipolytic enzymes, namely ATGL and HSL, were not changed following CEPM treatment during differentiation (data not shown).

As we know, mitochondria are the key organelles that produce cellular energy and control many processes from signal transmission to cell death. Mitochondrial dysfunction and oxidative stress have been extensively reported in obesity and diabetic patients (30), and there is an mTORC1-dependent increase in the production of mitochondrial complex III ROS during adipocyte differentiation (31). Lipid over-accumulation leads to high levels of intracellular ROS, which may exacerbate the oxidative process in obesity, and promote mitochondrial dysfunction (32–35). mtDNA is more susceptible to oxidative damage than nDNA due to its incomplete DNA repair capacity and the proximity of mtDNA to the respiratory chain. Our data showed that CEPM treatment greatly increased the mitochondrial content and copy number. CEPM also eliminated intracellular ROS in a dose-dependent manner, which would protect mitochondria from oxidative stress damage and maintain mitochondrial functional stability.

The study results also showed that CEPM strikingly elevated the mRNA expression levels of *Nanpt*, *Tafm*, *Nrf1,* and *Nrf2*, which are all involved in mitochondrial biogenesis. As has been described earlier, PGC1a is a positive regulator of mitochondrial biogenesis and respiration, adaptive thermogenesis, as well as many other metabolic processes (36). It activates nuclear respiratory factor 1 (NRF1), which is part of the communication between the nucleus and the mitochondrion, and triggers mitochondrial replication by the activation of mitochondrial transcription factor A (TFAM) (37, 38). Nicotinamide phosphoribosyl transferase (NAMPT) is a key enzyme in the biosynthetic pathway of nicotinamide adenine dinucleotide (NAD), which is an essential co-enzyme involved in cellular redox reactions. During various metabolic disorders, the level of NAD decreases, and thus, NAMPT regulates cell metabolism by affecting the activity of NAD-dependent enzymes (39). The nuclear respiratory factors, NRF1 and NRF2, are transcription factors that act on the majority of nuclear genes the products of which are required for respiratory chain expression and biological function (40).

UCP1 is considered to be the sole thermogenin responsible for adaptive non-shivering thermogenesis, and a high level of expression of UCP1 is a significant feature of BAT. PGC-1α can bind to various transcription factors and other transcription-related protein complexes to activate UCP1 and induce brown adipocytes to perform uncoupled respiration (41). The genes, *Cidea*, *Cd137,* and *Tbx1*, are widely considered as beige fat precursor markers (42), and their expression levels are increased with stimuli, such as zeaxanthin and melanocortins (42–45). In this study, CEPM drastically increased the mRNA expression levels of these three beige adipocyte-related biomarkers. In addition, the levels of several key browning-specific genes, including *Cox7a1*, *Cox8b*, *Pdk-4,* and *Cidea*, were significantly increased. The brown/beige adipocyte markers UCP1 and PGC1α were also significantly upregulated by CEPM treatment during differentiation. However, we did not use a myocyte cell line as a positive control for brown adipocyte. Primary brown adipocytes – the brown fat stromal vascular fraction (SVF) - have proved to be an excellent model. It can be easily obtained from BAT and can be induced to differentiate into mature adipocytes under appropriate conditions (46). Besides, several immortalised human and mouse brown adipocyte cell lines, such as T37i, WT-1, and HIB 1B, have been generated (47). Therefore, further studies are required to compare the brown adipocyte function between the beige adipocyte with CEPM treatment and the differentiated brown adipocyte.

Numerous studies have reported that plant extracts rich in flavonoids can induce the browning of white adipocytes, increase energy consumption, inhibit high-fat diet (HFD)-induced obesity and improve the metabolic status (25, 45, 48, 49). We assume that the flavonoids, such as naringin and hesperidin, as well as phenolic acids such as caffeic acid and citric acid in CEPM, partially contribute in promoting the browning/beiging of adipocytes, although further studies are required to explore this.

**Fig. 7:**
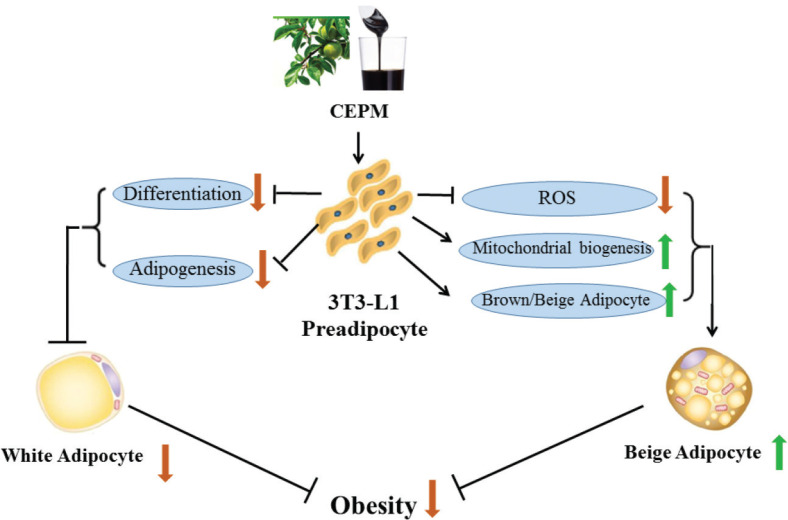
A schematic diagram of the molecular mechanism of CEPM’s anti-obesity effect in 3T3-L1 adipocytes.

Taken together, the results of this study reveal that CEPM elicits multiple effects, including ameliorating lipid metabolism, induction of browning/beiging marker genes, and stimulating mitochondrial biogenesis in 3T3-L1 cells (Fig. 7), all of which suggest that CEPM may be a useful therapeutic candidate for the treatment of obesity.
